# Interlayer Parallel Connection of Multiple Helmholtz Resonators for Optional Broadband Low Frequency Sound Absorption

**DOI:** 10.3390/ma18030682

**Published:** 2025-02-04

**Authors:** Xiaocui Yang, Qiang Li, Xinmin Shen, Binbin Zhou, Ning Wang, Enshuai Wang, Xiaonan Zhang, Cheng Shen, Hantian Wang, Shunjie Jiang

**Affiliations:** 1 Engineering Training Center, Nanjing Vocational University of Industry Technology, Nanjing 210023, China; 2019101052@niit.edu.cn (X.Y.); 2020101101@niit.edu.cn (B.Z.); 2021101227@niit.edu.cn (N.W.); 2017100884@niit.edu.cn (H.W.); 2022301363@niit.edu.cn (S.J.); 2 MIIT Key Laboratory of Multifunctional Lightweight Materials and Structures (MLMS), Nanjing University of Aeronautics and Astronautics, Nanjing 210016, China; wangenshuai0823@126.com (E.W.); cshen@nuaa.edu.cn (C.S.); 3 The 28th Research Institute of China Electronics Technology Group Corporation, Nanjing 210007, China; 4 College of Field Engineering, Army Engineering University of PLA, Nanjing 210014, China; zxn8206@163.com

**Keywords:** acoustic metamaterial, interlayer parallel connection, Helmholtz resonator, sound absorption performance, finite element simulation, sound absorption mechanism, optimization

## Abstract

The Helmholtz resonance acoustic metamaterial is an effective sound absorber in the field of noise reduction, especially in the low-frequency domain. To overcome the conflict between the number of Helmholtz resonators and the volume of the rear cavity for each chamber with a given front area of single-layer metamaterial, a novel acoustic metamaterial of interlayer parallel connection of multiple Helmholtz resonators (IPC–MHR) is proposed in this study. The developed IPC–MHR consists of several layers, and the Helmholtz resonators among different layers are connected in parallel. The sound absorption property of IPC–MHR is studied by finite element simulation and further optimized by particle swarm optimization algorithm, and it is validated by standing wave tube measurement with the sample fabricated by additive manufacturing. The average sound absorption coefficient in the discrete frequency band [200 Hz, 300 Hz] U [400 Hz, 600 Hz] U [800 Hz, 1250 Hz] is 0.7769 for the IPC–MHR with four layers. Through the optimization of the thickness of each layer, the average sound absorption coefficient in 250–750 Hz is up to 0.8068. Similarly, the optimized IPC–MHR with six layers obtains an average sound absorption coefficient of 0.8454 in 300–950 Hz, which exhibits an excellent sound absorption performance in the low-frequency range with a wide band. The IPC–MHR can be used to suppress obnoxious noise in practical applications.

## 1. Introduction

With the ongoing enhancement of the living standard, the individuals’ expectations regarding acoustic environments have significantly increased [1,2]. This is particularly evident in metropolitan areas, where the conflict between pervasive noise pollution and the public’s demand for tranquil living conditions has become increasingly pronounced [3,4,5,6]. Rosario–Gilabert et al. [1] demonstrated the potential benefits of utilizing recycled acoustic panels from plastic bottles and PET felt composites in healthcare environments to suppress noise and improve acoustic comfort. With the well-established importance of healthy indoor acoustic environments, the investigations on how occupants evaluate, understand, and characterize healthy indoor acoustic environments in residential buildings were conducted by Chen et al. [2]. As a first step to tackling the problem of deteriorated urban soundscapes, Bonet-Solà et al. [3] aimed to develop a tool that automatically evaluated the soundscape quality of dwellings based on the acoustic events obtained from short videos recorded on-site. Vasconcelos et al. [4] investigated the effects of noise exposure for 24 h on young adults and old zebrafish, which was the first attempt to investigate the effects of both noise and aging on zebrafish behavior, suggesting the age-dependent physiological coping mechanisms associated with environmental stress. Song et al. [5] examined the control of traffic noise in urban planning, considering the differences in noise impacts at various scales, which investigated the noise impact in urban centers and fringes and analyzed varying effects of a given variable on traffic noise at the scales of 300, 600, and 1200 m. Based on noise complaint data, the quantitative analysis of the perceived noise environment in New York City was conducted by Chen et al. [6], which could serve as a reference for the construction of noise-friendly high-density cities and the promotion of sustainable urban health.

The noise with changeful spectrum ranges and the limitation of available space put forward higher requirements for the sound-absorbing materials, especially in the low-frequency domain [7,8], making it difficult to obtain the balance between desired sound absorption bandwidth and actual occupied space [9,10,11,12,13,14,15,16]. It was pointed out by Yu et al. [7] that the low-frequency discrete variable noise sources were frequently encountered in daily life, and traditional acoustic structures faced challenges in effectively absorbing such variable noise below 600 Hz due to the variability in noise frequency and the inherent characteristics of longer wavelengths. For example, the road noise varies with the vehicles’ load and speed, ranging from low to medium frequencies, and the existing absorbers cannot fully mitigate the variable noise due to limited bandwidth [8]. Xu et al. [9] investigated the acoustic noise emissions from a direct torque-controlled induction motor drive, and it was proved that both the bandwidths of the flux and torque hysteresis controllers and the equivalent modulation indices could have a significant influence on the switching frequency and the spread spectrum of the harmonic content. It was pointed out by Ma and Ye [12] that environmental noise pollution was a growing challenge worldwide, necessitating effective sound absorption strategies to improve acoustic environments, and the materials that drew inspiration from nature’s structural design principles could provide enhanced functionalities. With the rapid development of the traffic industry, noise issues are becoming increasingly serious, and traditional noise control technologies have the problems of poor low-frequency noise absorption and narrow bandwidth [13]. Yang et al. [14] developed the twenty-four-hour noise maps of the Chancheng District in Foshan, China, which certified that it was urgent to develop effective noise reduction measures to mitigate traffic noise pollution at night based on the evaluation result. The characteristics of the airborne and structure-borne noise generated by these infrastructures were studied by Chen et al. [16], and the variation in the outdoor road noise across different floors over the entire frequency range demonstrated an initial increase followed by a decrease with rising floor height due to air damping effects.

Therefore, many tunable acoustic metamaterials have been developed to attempt to make the sound absorption band of the acoustic metamaterials adjustable according to the change in noise spectrum [17,18,19,20,21,22,23,24,25,26,27,28,29,30], while being able to take up as little space as possible and have simple structures. Wang et al. [17] introduced the folded slit to enhance the micro-slit acoustic absorber, effectively improving its low-frequency sound absorption performance and successfully achieving a perfect acoustic absorption coefficient of 0.99 at a thickness of only 3.1 cm. Saatchi et al. [20] proved that the parametric design of alternating unit cells of Schwarz primitive triply periodic minimal surface metamaterial could offer the capability to create both single tunable bandgap and multiple bandgaps. Chen et al. [21] introduced an origami-based acoustic metamaterial structure that consisted of a Miura-ori foldcore, along with a perforated and an unperforated panel, which showed adjustable low-frequency sound absorption capacities due to the foldability of origami foldcore. A novel dual-target locally resonant acoustic metamaterial was proposed by Ravanbod et al. [24] based on the coupling cavity and convex mechanisms, utilizing the benefits of both sound-barrier and sound-absorbing types of acoustic metamaterials. An acoustic metamaterial consisting of Helmholtz resonators with porous material lining was proposed by Zhang and Xin [25] to gain broadband low-frequency sound absorption, and a good tunable sound absorption could be achieved by varying the length and diameter of the embedded neck. Bi et al. [28] developed a stackable and expandable acoustic metamaterial with multiple tortuous channels, which consisted of odd panels, even panels, chambers, and final closing plate, and these component parts could be fabricated separately and then assembled. An online tunable acoustic metamaterial with an optional aperture and adjustable cavity was proposed by Yang et al. [30], and its sound absorption properties were preliminarily optimized by the joint three-dimensional finite element simulation model and particle swarm optimization algorithm.

Based on these conditions [17,18,19,20,21,22,23,24,25,26,27,28,29,30], a novel acoustic metamaterial of interlayer parallel connection of multiple Helmholtz resonators (IPC–MHR) is proposed in this study, aiming to obtain an optional broadband sound absorption in the low-frequency domain. First, the overall structure of IPC–MHR metamaterial is divided into some superposable layers, and each layer contains multiple Helmholtz resonators, and all Helmholtz resonators are in parallel connection by the clever design. Second, the acoustic finite element simulation model of single-group IPC–MHR and that of the multi-group IPC–MHR are constructed, respectively, which can show the action principle of the proposed IPC–MHR metamaterial. Meanwhile, according to the distributions of the viscous power densities at these peak sound absorption frequency points for the multi-group IPC–MHR, the sound absorption mechanism is visually displayed. Afterward, the samples for these layers are fabricated by additive manufacturing, and they are assembled to form the proposed IPC–MHR metamaterial, its actual sound absorption coefficient is tested by a standing wave tube to verify the feasibility of this novel design, and the accuracy of the utilized acoustic finite element simulation. Moreover, the sound absorption performances of the IPC–MHR acoustic metamaterial are further improved by the optimization of parameters, the adjustment of the thickness of each layer, and the increase in the number of layers, respectively, which are conducive to promoting its practical applications in noise suppression.

## 2. Structural Design

**Figure 1:**
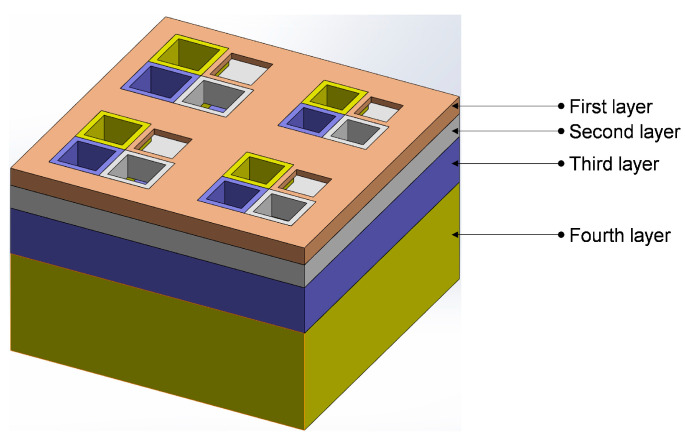
The overall structure of the proposed IPC–MHR acoustic metamaterial.

**Figure 2:**
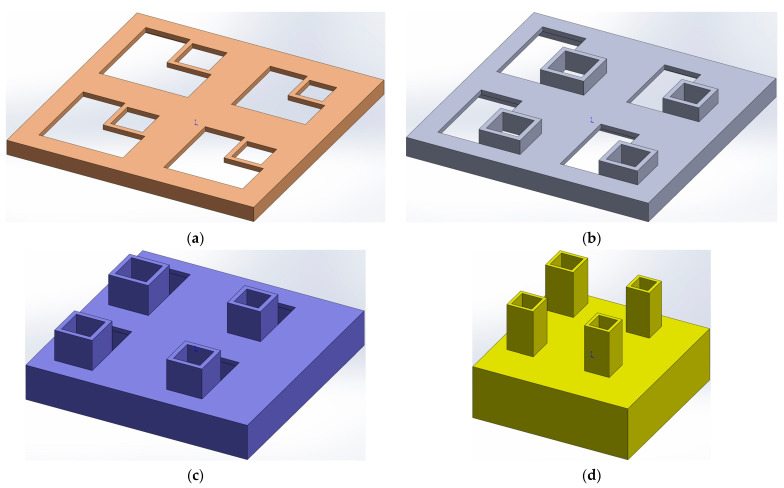
The structure of each layer in the IPC–MHR acoustic metamaterial. (**a**) The first layer; (**b**) the second layer; (**c**) the third layer; and (**d**) the fourth layer.

The overall structure of the proposed IPC–MHR acoustic metamaterial with four layers is shown in Figure 1, and the corresponding four single layers are shown in Figure 2a–d, respectively. It can be found that there are 4 Helmholtz resonators in each layer, which indicates that there are 16 Helmholtz resonators in parallel connections in the IPC–MHR. It should be specially noted that the Helmholtz resonators among the different layers are connected in parallel rather than in series by the appropriate design. Taking the first layer shown in Figure 2a, for example, except for the square aperture for the incident acoustic wave, the other breaches on the front panel are filled up by corresponding square incident apertures in the back layers. Thus, the IPC–MHR acoustic metamaterial adopts a cascading method from back to front, and the corresponding sound absorption property can be adjusted by adding or removing the layers from front to back. Moreover, the symbols used in this study and their corresponding meanings are summarized in Appendix A.

**Figure 3:**
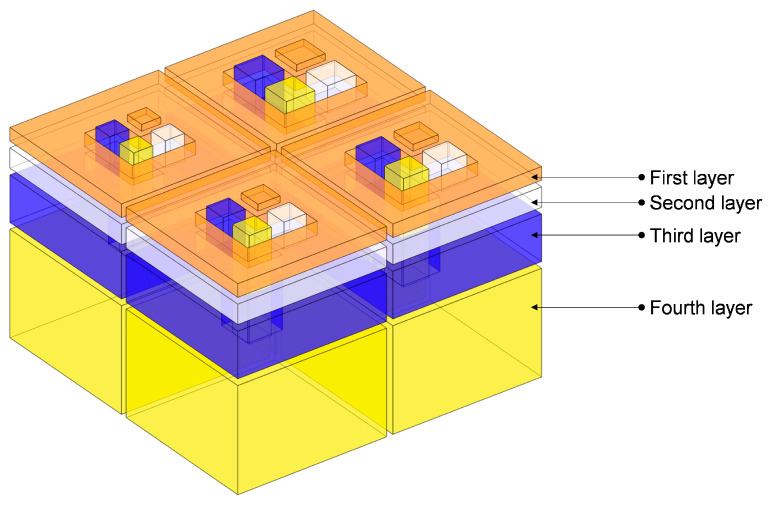
The air domain of the proposed IPC–MHR acoustic metamaterial.

**Figure 4:**
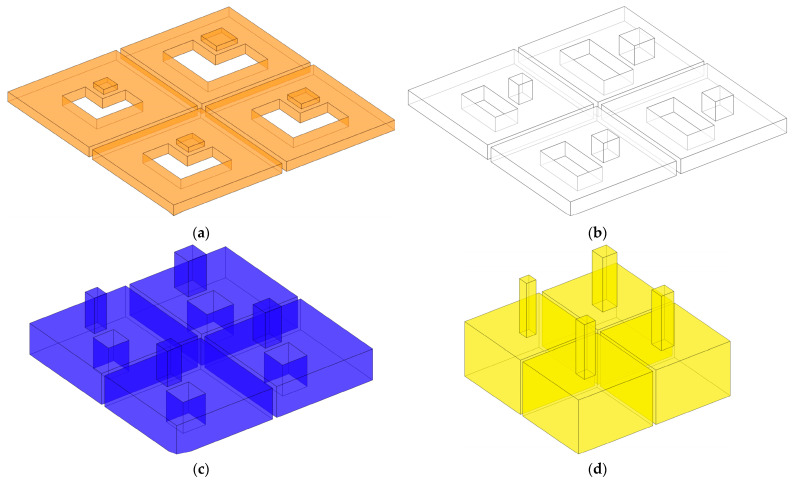
The air domain of each layer in the IPC–MHR acoustic metamaterial. (**a**) The first layer; (**b**) the second layer; (**c**) the third layer; and (**d**) the fourth layer.

To better demonstrate the structural composition of IPC–MHR acoustic metamaterial, its corresponding air domain is shown in Figure 3, and that air domain for each layer is exhibited in Figure 4. It can be shown more intuitively that IPC–MHR acoustic metamaterial is composed of multiple Helmholtz resonators and these Helmholtz resonators among different layers are connected in parallel. Although the 4 layers in IPC–MHR acoustic metamaterial are physically connected in series, the 16 Helmholtz resonators are connected in parallel, because all the square incident apertures are connected to the front to receive incoming sound waves with various frequencies. Meanwhile, the parallel connection of multiple Helmholtz resonators stacked in one layer for the acoustic metamaterial in the literature makes it difficult to obtain broadband sound absorption in the low-frequency band, because there is an irreconcilable conflict between the number of Helmholtz resonators and the volume of the rear cavity for each chamber with given front area of the acoustic metamaterial [17,18,19,20,21,22,23,24,25,26,27,28,29,30]. If the acoustic metamaterial is divided into too many Helmholtz resonators, although the sound absorption bandwidth can be enlarged, it is difficult to realize in the low-frequency band because the volume of the rear chamber for each chamber is small. On the contrary, if the volume of the rear chamber for each chamber is enlarged, the sound absorption in the low-frequency band can be obtained, but the bandwidth will be significantly reduced because the number of Helmholtz resonators is decreased when the total front area is limited and the bandwidth generated by the single Helmholtz resonator is small. Fortunately, the proposed IPC–MHR acoustic metamaterial can overcome this contradiction. The increase in the number of Helmholtz resonators by adding the layers hardly affects the volume of the individual chambers in each layer, and the volume of Helmholtz resonators in each layer can be adjusted flexibly by tuning the corresponding thickness. Moreover, although the IPC–MHR acoustic metamaterial in Figure 1 and Figure 3 has four layers, its number of layers can be increased or decreased as needed. Furthermore, the number of Helmholtz resonators in each layer can be tuned as well, which can meet the needs of different bandwidths in various frequency segments.

According to the acoustoelectric analogy method of the Helmholtz resonator [31,32,33,34,35], the theoretical sound absorption coefficient α of the IPC–MHR acoustic metamaterial can be derived by Equation (1). Here *Z_total_* is the total acoustic impedance of the IPC–MHR acoustic metamaterial and *Z*_0_ is the acoustic impedance of air under the condition of normal temperature and pressure, 415 Kg/(m^2^·s).

(1) $$\alpha =1-{\left|\frac{Z_{total}-Z_{0}}{Z_{total}+Z_{0}}\right|}^{2}$$

Though the *Z_total_* can be derived based on the Helmholtz resonance principle [36,37,38], the prediction accuracy of the theoretical model is unsatisfactory because of the following several reasons. First, the normal Helmholtz resonance principle is derived from classical Helmholtz resonators with a cylindrical aperture and cylindrical chamber, but the front incident aperture in the IPC–MHR acoustic metamaterial is cuboid, and the corresponding rear chamber is an irregular shape, which indicates that some approximation and equivalence are inevitable. Second, in the classical Helmholtz resonator, the front aperture and the rear chamber are coaxial, but the relative positions of the two in Figure 1, Figure 2, Figure 3 and Figure 4 are eccentric, which will inevitably result in some error. Third, the length of the aperture is an important factor to affects the prediction accuracy, and it should be in a reasonable range, but the length is too small in the first layer, and it is too large in the fourth layer, which further reduces the accuracy of the theoretical model. Meanwhile, there are some essential simplifications, omissions, approximations, and equivalences in the theoretical modeling process, which will lead to a large deviation between the prediction results of the theoretical model and the experimental results. Therefore, in many studies, the theoretical model is a basement to study the feasibility of a novel acoustic metamaterial, and the acoustic finite element simulation is treated as a more effective way to predict the sound absorption coefficient and intuitively show its sound absorption mechanism [39,40,41].

## 3. Acoustic Finite Element Simulation

**Figure 5:**
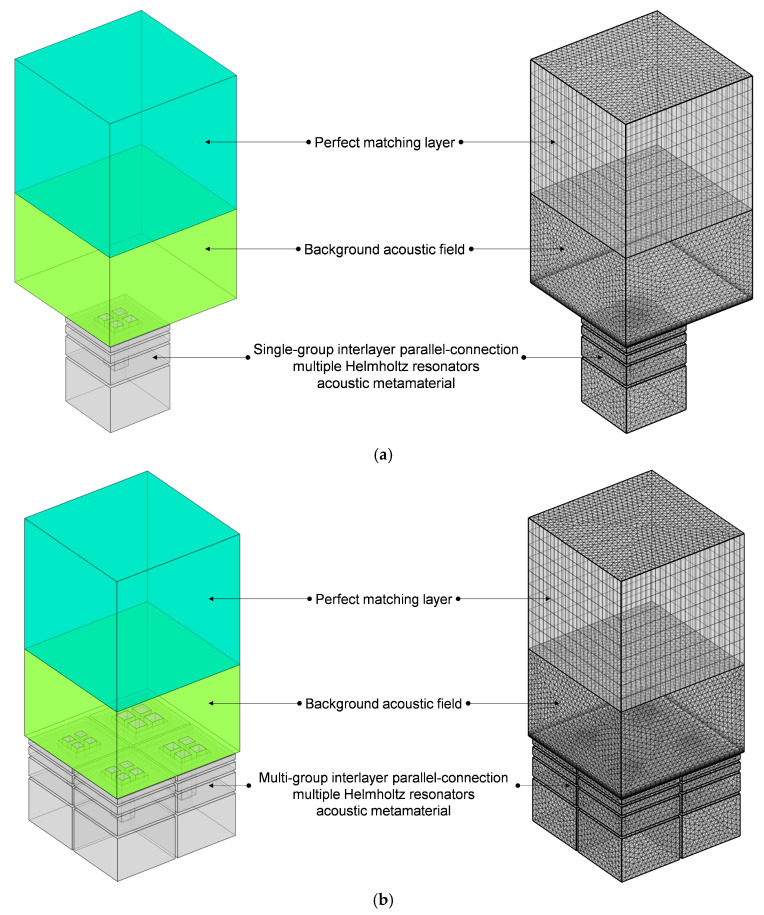
The acoustic finite element simulation of the proposed IPC–MHR acoustic metamaterial. (**a**) The single-group IPC–MHR; and (**b**) the multi-group IPC–MHR.

The finite element simulation model of the proposed IPC–MHR acoustic metamaterial is shown in Figure 5. For single-group IPC–MHR in Figure 5a, the side length of the square incident aperture is equal, which is symbolled as a_i_ (i = 1, 2, 3, 4) for these four modules in Figure 5b. Meanwhile, the total thickness of each layer is symbolled as T_i_ (i = 1, 2, 3, 4), and the thickness of all the walls is symbolled by t_0_ with a uniform value of 2 mm. Thus, it can be calculated that the length of the aperture is t_0_, T_1_, T_2_, and T_3_, respectively, for the four layers, and the corresponding thickness of the rear cavity is T_1_–t_0_, T_2_–T_1_–t_0_, T_3_–T_2_–t_0_, and T_4_–T_3_–t_0_, respectively. Meanwhile, the meanings of these symbols are applicable to the multi-group IPC–MHR in Figure 5b as well. Moreover, taking the following standing wave tube measurement into consideration, the cross-sectional shape of the multi-group IPC–MHR in Figure 5b is square with a side length of S_0_ = 100 mm, and its total thickness is T_4_ + t_0_. For the single-group IPC–MHR in Figure 5a, its side length is (S_0_ − 3t_0_)/2. Normally speaking, the length of the front aperture should be no more than the thickness of the rear cavity. Therefore, in the initial IPC–MHR, the thickness of each layer is set as T_1_ = 3t_0_, T_2_ = 2T_1_ + t_0_, T_3_ = 2T_2_ + t_0_, and T_4_ = 2T_3_ + t_0_, respectively. Except for the first layer, the length of the front aperture for the other three layers is equal to the thickness of the corresponding rear cavity for this investigated IPC–MHR acoustic metamaterial.

All the structural parameters for IPC–MHR acoustic metamaterial in Figure 5 are summarized in Table 1. The total thickness of the IPC–MHR is equal to the thickness of the fourth layer T_4_ combined with that of the final back panel t_0_. It should be noted that the parameters for the initial IPC–MHR metamaterial have definite interrelationships among the thickness of each layer, but these constraint conditions are not necessary for the subsequent studies. Similarly, the side length of the square incident aperture for the multi-group IPC–MHR can be adjusted to obtain the desired sound absorption performance in the expected frequency range. Moreover, the number of layers in the IPC–MHR acoustic metamaterial can be altered too, which can tune the effective frequency band.

Meanwhile, all the used finite element simulation parameters for IPC–MHR acoustic metamaterial in Figure 5 are summarized in Table 2, and they apply to the subsequent finite element simulation process as well except the investigated frequency range, which is altered with the actual needs of various simulations. The thickness of the background field is 64 mm, which is equal to the total thickness of the investigated IPC–MHR acoustic metamaterial, and that of the perfect matching layer is 1.5 times it. The selection of these parameters is to improve the simulation accuracy in the low-frequency range as much as possible. Generally speaking, the finer the meshing grid is, the better the simulation accuracy will be, but the computational workload will be greatly increased as well. Thus, the parameters for the meshing grid, such as the curvature factor, number of layers in the boundary, etc., are selected by taking into account both efficiency and precision.

**Figure 6:**
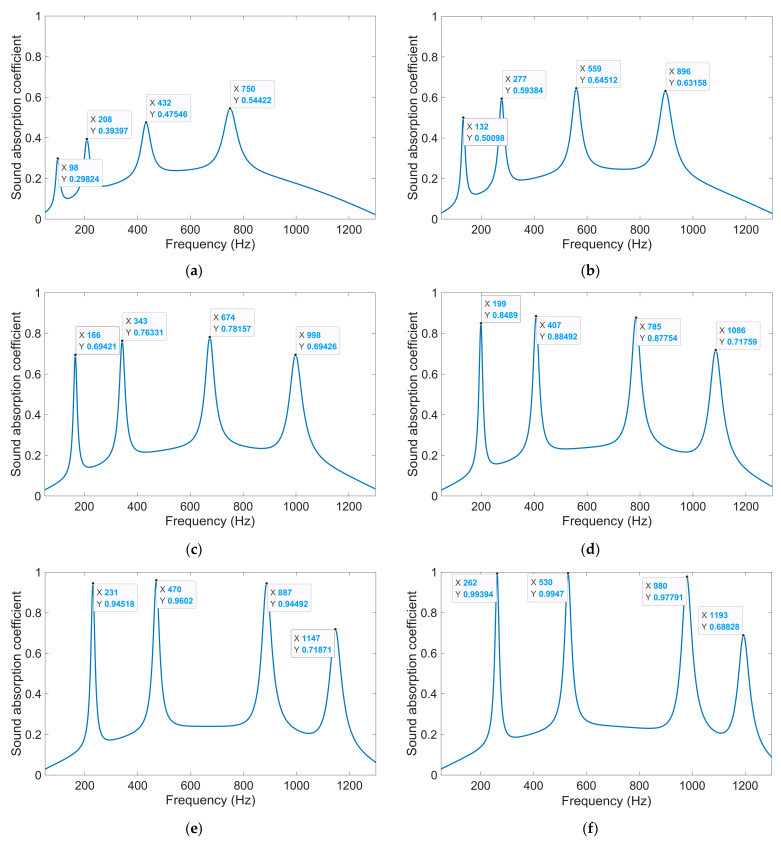

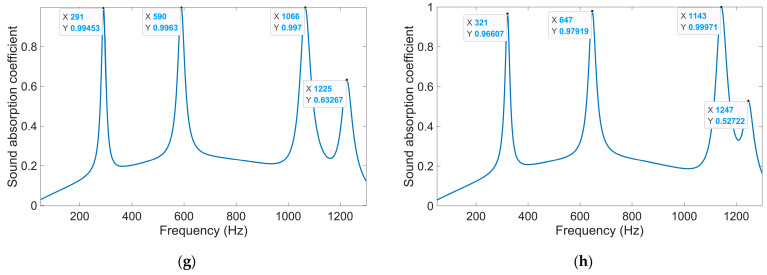
The distributions of sound absorption coefficients for the single-group IPC–MHR with the various side lengths of the square incident aperture: (**a**) 3 mm; (**b**) 4 mm; (**c**) 5 mm; (**d**) 6 mm; (**e**) 7 mm; (**f**) 8 mm; (**g**) 9 mm; and (**h**) 10 mm.

The distributions of sound absorption coefficients for single-group IPC–MHR in Figure 5a are exhibited in Figure 6a–h corresponding to the various side lengths of the square incident aperture ranging from 3 mm to 10 mm with the interval of 1 mm, respectively. It can be judged from Figure 6a,b that though the resonance frequency can be small when the side length of the square incident aperture is small, the corresponding peak sound absorption coefficient is too low, which makes it lose practical significance. Meanwhile, it can be judged from Figure 6g,h that when the side length of the square incident aperture is large, the fourth peak sound absorption coefficient is too low as well, which makes it lose the practical value, too. Thus, it can be proved that the side length of the square incident aperture should be in a reasonable range, such as the four satisfactory peak sound absorption coefficients for each condition in Figure 6c–f. Moreover, the sound absorption property of each condition is different, which means that the desired sound absorption performance can be achieved by selecting the suitable combination of the different single-group IPC–MHR to form the multi-group IPC–MHR.

**Figure 7:**
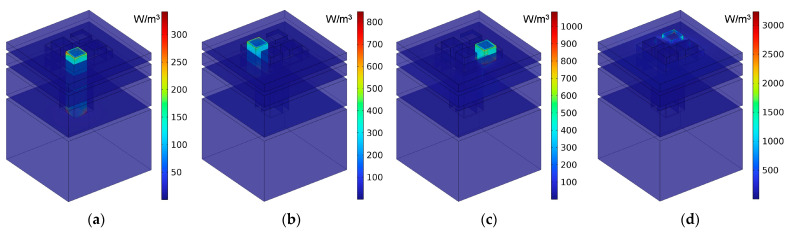
The distributions of the viscous power densities at the four resonance frequencies when the side length of the square incident aperture for the single-group IPC–MHR is 7 mm: (**a**) 231 Hz; (**b**) 470 Hz; (**c**) 887 Hz; and (**d**) 1147 Hz.

Taking the single-group IPC–MHR with the side length 7 mm of the square incident aperture for example, the distributions of the viscous power density at four resonance frequencies of 231 Hz, 470 Hz, 887 Hz, and 1147 Hz are obtained, which are shown in Figure 7a–d, respectively. It can be found that each layer can contribute a resonance frequency independently, and the sound absorption effect is mainly realized by the thermal viscosity effect in the incident aperture, especially at the location of walls of the incident aperture. It should be noted that the final sound absorption coefficient for a certain frequency point is decided by the distributions of viscous power densities and the corresponding effective volume collectively. That is why the corresponding sound absorption coefficients of these four resonance frequencies are 0.9452, 0.9602, 0.9449, and 0.7187, respectively.

**Figure 8:**
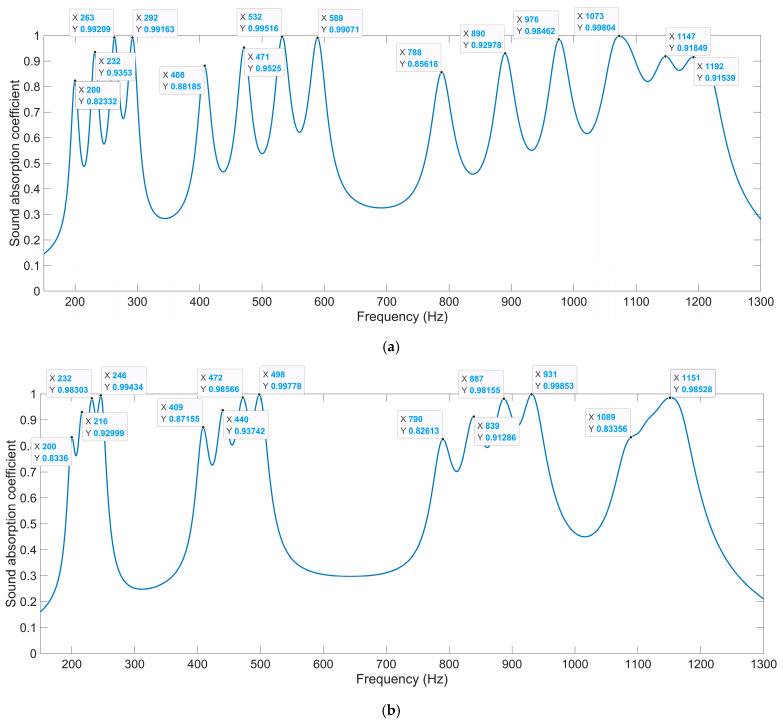
The distributions of sound absorption coefficients for the multi-group IPC–MHR with the various combinations of side length of the square incident aperture for the 4 modules: (**a**) 6 mm + 7 mm + 8 mm + 9 mm; and (**b**) 6 mm +6.5 mm + 7 mm + 7.5 mm.

**Figure 9:**
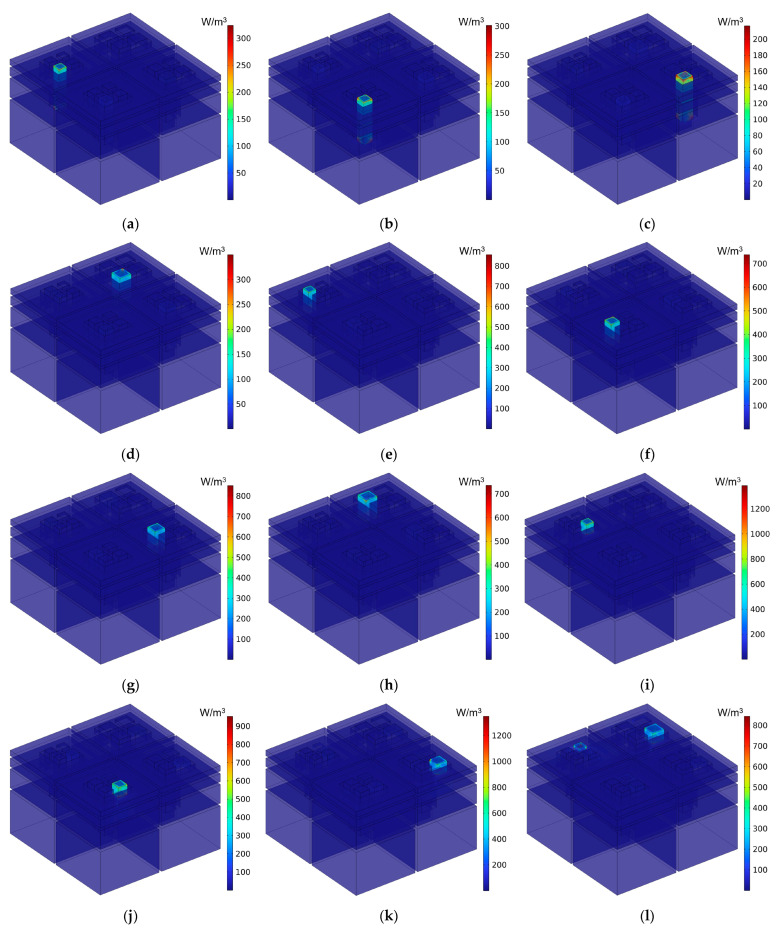

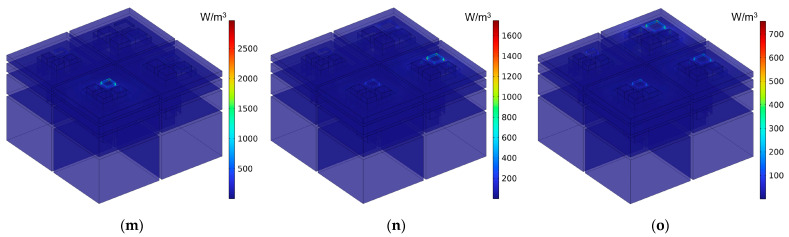
The distributions of viscous power densities at 15 frequency points for the multi-group IPC–MHR when the side length of the square incident aperture for the 4 modules are 6 mm, 7 mm, 8 mm, and 9 mm: (**a**) 200 Hz; (**b**) 232 Hz; (**c**) 263 Hz; (**d**) 292 Hz; (**e**) 408 Hz; (**f**) 471 Hz; (**g**) 532 Hz; (**h**) 589 Hz; (**i**) 788 Hz; (**j**) 890 Hz; (**k**) 976 Hz; (**l**) 1073 Hz; (**m**) 1147 Hz; (**n**) 1192 Hz; and (**o**) 1250 Hz.

The distributions of the sound absorption coefficients of the multi-group IPC–MHR when the side length of the square incident aperture for the four modules are 6 mm, 7 mm, 8 mm, and 9 mm are shown in Figure 8a. It can be found that there are four groups of sound absorption peaks generated in the frequency ranges 150–300 Hz, 400–600 Hz, 750–1100 Hz, and 1100–1250 Hz, respectively. To further exhibit the sound absorption characteristics of the multi-group IPC–MHR, the distributions of the sound absorption coefficients when the side length of the square incident aperture for the four modules are 6 mm, 6.5 mm, 7 mm, and 7.5 mm are obtained by the acoustic finite element simulation model in Figure 5b too, as shown in Figure 8b. Comparing the distribution of sound absorption coefficients in Figure 8a and that in Figure 8b, it can be found that the sound absorption peaks are closer in each discrete sound absorption band when the side length of the square incident aperture for the four modules is nearer. Meanwhile, it is interesting to note that the sound absorption property of each module is almost independent. In Figure 6d, the four resonance frequencies for single-group IPC–MHR with the side length of the square incident aperture of 6 mm are 199 Hz, 407 Hz, 785 Hz, and 1086 Hz. Accordingly, the sound absorption peaks contributed by the module with the side length of the square incident aperture of 6 mm in Figure 8a are 200 Hz, 408 Hz, 788 Hz, and 1073 Hz (coupling with the third resonance frequency of the module with the side length of the square incident aperture of 9 mm), and those in Figure 8b are 200 Hz, 409 Hz, 790 Hz, and 1089 Hz (the coupling effect is similar with that in Figure 8a). It can be found that the absorption effect of each module is relatively independent, especially in the low-frequency bands. The distributions of the viscous power densities for the 15 representative frequencies in Figure 8a are shown in Figure 9, which can further prove this inference.

## 4. Results and Discussions

### 4.1. Experimental Validation

**Figure 10:**
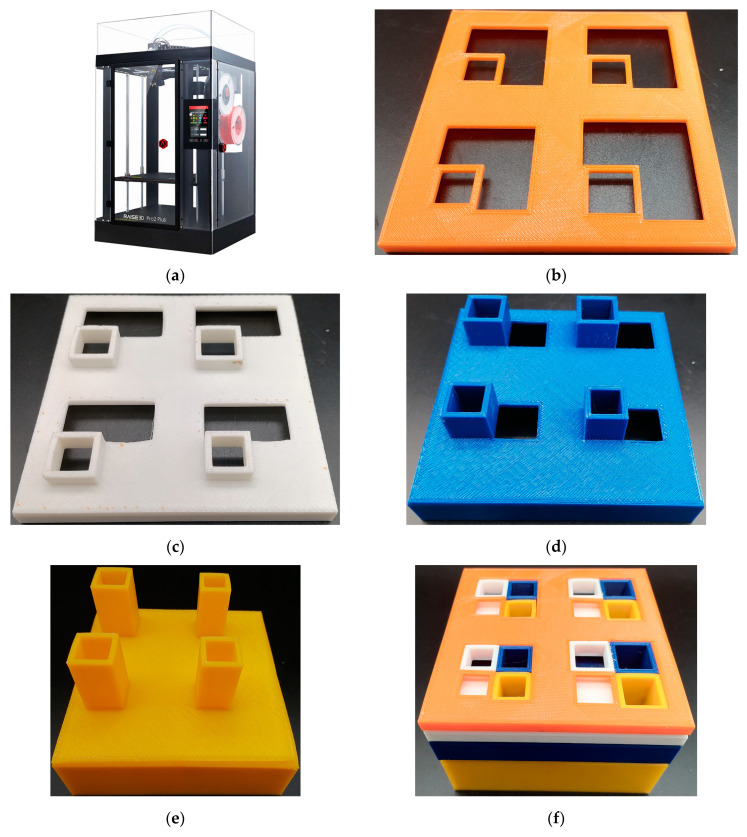
The fabrication of the multi-group IPC–MHR when the side length of the square incident aperture for the 4 modules are 6 mm, 7 mm, 8 mm, and 9 mm. (**a**) The utilized additive manufacturing machine; (**b**) the first layer; (**c**) the second layer; (**d**) the third layer; (**e**) the fourth layer; and (**f**) the assembled multi-group IPC–MHR sample.

To verify the feasibility of this design and the accuracy of the finite element simulation model, when the side length of the square incident aperture for four modules are 6 mm, 7 mm, 8 mm, and 9 mm, the samples of four layers for multi-group IPC–MHR are fabricated by additive manufacturing shown in Figure 10a (Raise 3D Pro2 Plus, Raise3D, Shanghai, China, https://www.raise3d.cn), and the obtained four samples for each layer are shown in Figure 10b–e, respectively. Afterward, the four layers are assembled to form the proposed multi-group IPC–MHR metamaterial, as shown in Figure 10f. The other structural parameters for this fabricated sample are consistent with those summarized in Table 1. It can be observed that the multi-group IPC–MHR can be assembled from the fourth layer to the first layer one by one, and there are four modules in each layer. Moreover, the assemblies of third + fourth and second + third + fourth can act as sound absorbers independently as well, which can modify the effective frequency band simply.

**Figure 11:**
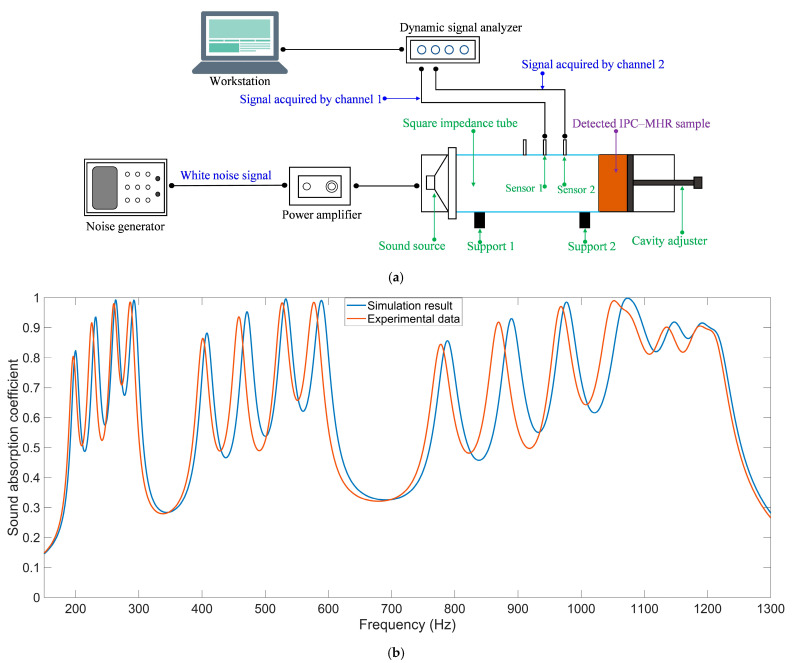
The experimental validation of sound absorption performance of the multi-group IPC–MHR. (**a**) The utilized standing wave tube system; and (**b**) the comparisons of the sound absorption coefficients between simulation result and experimental data.

Afterward, the actual sound absorption coefficients of the assembled multi-group IPC–MHR sample are tested by a standing wave tube shown in Figure 11a [42,43,44] (F100/F50 standing wave tube system, BSWA TECHNOLOGY CO., LTD., Beijing, China, http://www.bswa-tech.com), and the comparisons of experimental data with simulation results are shown in Figure 11b. It can be found that the consistency is satisfactory, which proves the effectiveness of this IPC–MHR and the accuracy of the finite element simulation model. Meanwhile, it can be found that the experimental data shifts to the low-frequency direction slightly relative to the simulation result. The major reason for this phenomenon is the existing edge expansion effect in the additive manufacturing process to fabricate the sample, leading to a slight decrease in the diameter of the aperture and a minor increase in the length of the aperture, which results in the shift of the resonance frequency of each Helmholtz resonator in the proposed IPC–MHR acoustic metamaterial to the low-frequency direction.

### 4.2. Further Investigations and Discussions

It can be judged from the distributions of sound absorption coefficients of the investigated IPC–MHR acoustic metamaterial in Figure 8 that sound absorption property can be adjusted by adjusting the structural parameters. Thus, further investigations and discussions are conducted to obtain the expected sound absorption performance by the following methods, which include parametric optimization, optimization of the thickness of each layer, and the alteration of the number of layers.

#### 4.2.1. Parametric Optimization

**Figure 12:**
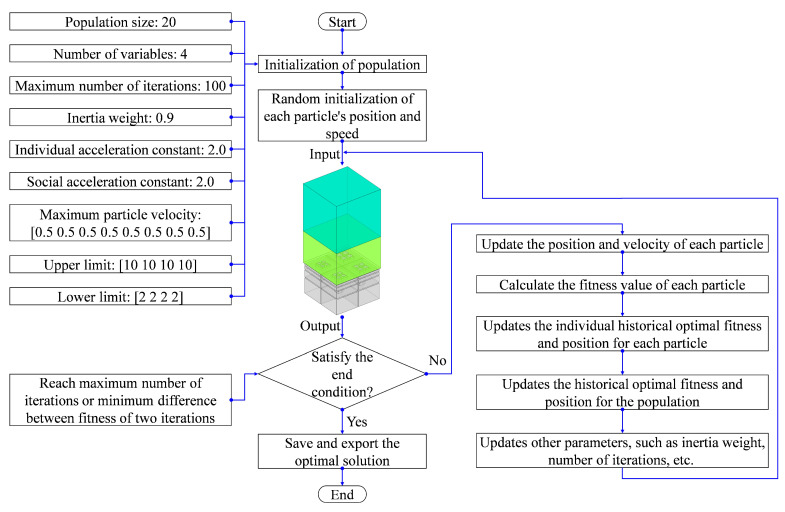
The flow chart of the particle swarm optimization algorithm utilized in this research and the corresponding selected parameters.

**Figure 13:**
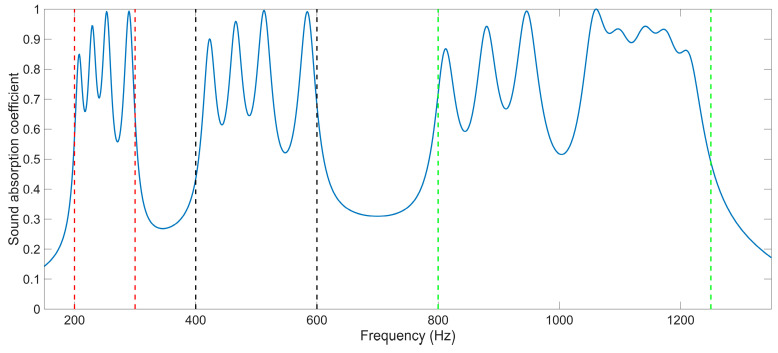
The distribution of optimal sound absorption property for the expected combinations of frequency domains 200–300 Hz, 400–600 Hz, and 800–1250 Hz.

The combinations of side lengths of the square incident aperture for the four modules in the multi-group IPC–MHR acoustic metamaterial can be optimized to obtain the expected sound absorption performance. Among these normal optimization algorithms, the particle swarm optimization algorithm is selected to optimize these structural parameters in this research, because it has the advantages of being simple and easy to implement, having fast convergence speed, and few parameters, while it has been applied in the actual parametric optimization of various acoustic metamaterials [45,46,47,48]. The flow chart of the particle swarm optimization algorithm utilized in this study and the corresponding selected parameters are shown in Figure 12. The available value range of each side length of the square incident aperture for the 4 modules is [3 mm, 10 mm]. Taking the expected combinations of frequency domains 200–300 Hz, 400–600 Hz, and 800–1250 Hz for example, the optimization objective is to obtain the maximum average sound absorption coefficient in the discrete frequency band. The optimal parameters of the side length of the square incident aperture for the four modules are 6.3 mm, 6.8 mm, 7.9 mm, and 8.9 mm, and the corresponding distribution of the optimal sound absorption coefficient is shown in Figure 13. The average sound absorption coefficient in the discrete frequency band [200 Hz, 300 Hz] U [400 Hz, 600 Hz] U [800 Hz, 1250 Hz] is 0.7769, and that for each frequency band is 0.7751 for 200–300 Hz, 0.7408 for 400–600 Hz, and 0.7935 for 800–1250 Hz, respectively. The first resonance frequency is achieved at 208 Hz with the corresponding sound absorption coefficient of 0.8499, and the total thickness of the proposed multi-group IPC–MHR acoustic metamaterial is 64 mm, which indicates that the thickness is only 1/26 of the wavelength of the incident acoustic wave corresponding to 208 Hz (0.064 × 208/343 ≈ 1/26).

#### 4.2.2. Optimization of Thickness of Each Layer

**Figure 14:**
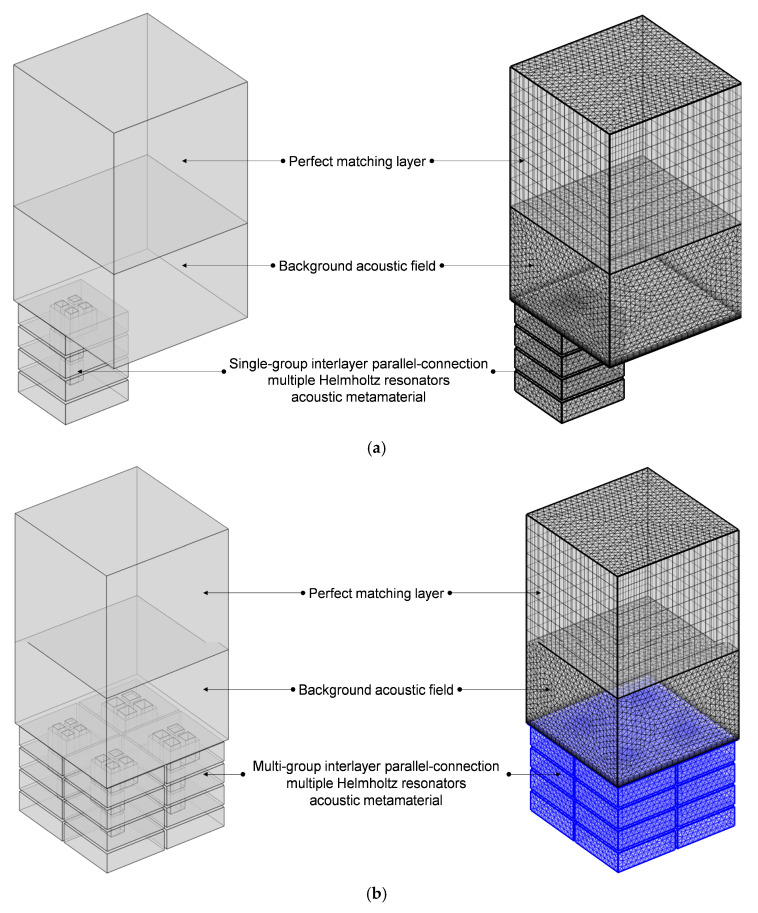
The finite element simulation of IPC–MHR acoustic metamaterial for the expected frequency range 250–750 Hz. (**a**) The single-group IPC–MHR; and (**b**) the multi-group IPC–MHR.

**Figure 15:**
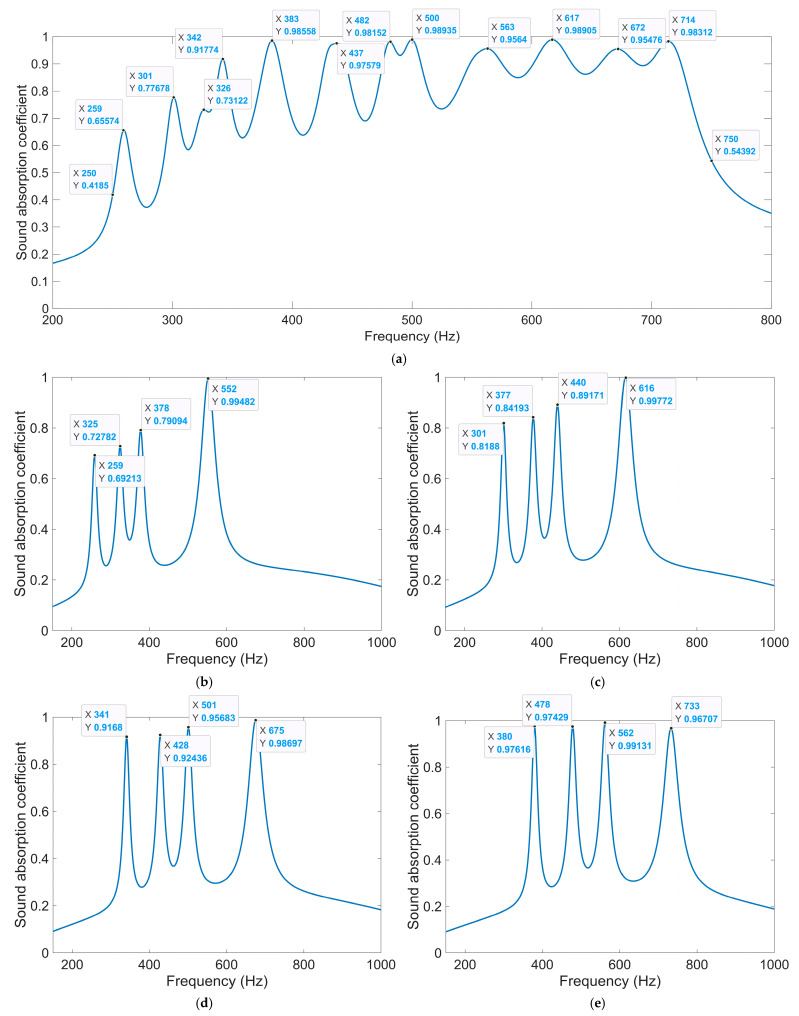
The distribution of optimal sound absorption property for the expected frequency range of 250–750 Hz. (**a**) The overall sound absorption performance; (**b**) the first module; (**c**) the second module; (**d**) the third module; and (**e**) the fourth module.

Except for the side length of the square incident aperture for the four modules, the thickness of each layer can be adjusted to achieve the desired sound absorption performance in the expected frequency range as well. Supposing the expected frequency range is 250–750 Hz, the thicknesses of four layers are optimized by the particle swarm optimization algorithm in Figure 12 when the side length of the square incident aperture for the four modules are set as 6 mm, 7 mm, 8 mm, and 9 mm. The allowed ranges of values of the thicknesses of four layers T_1_, T_2_, T_3_, and T_4_ are [2t_0_, T_2_], [4t_0_, T_3_], [6t_0_, T_4_] and [8t_0_, 62], respectively, which aim to keep the total thickness of acoustic metamaterial same with that of the IPC–MHR acoustic metamaterial in Figure 8. The utilized finite element simulation of IPC–MHR acoustic metamaterial for the expected frequency range 250–750 Hz is shown in the Figure 14. The obtained optimal thicknesses of four layers are 26 mm, 38 mm, 49 mm, and 62 mm, respectively, and the distributions of the sound absorption coefficients are shown in Figure 15a. The average sound absorption coefficient in 250–750 Hz is up to 0.8068, and there are 12 sound absorption peaks in this frequency range. Meanwhile, the sound absorption properties of four modules with optimal thicknesses of four layers are gained by the finite element simulation model in Figure 14a, and the distributions of sound absorption coefficients are exhibited in Figure 15b–e, respectively. The distributions of these sound absorption peaks are consistent between single-group IPC–MHR and multi-group IPC–MHR, especially in the low-frequency range, and the four lost sound absorption peaks are due to coupling effects among different resonators.

#### 4.2.3. Alteration of Number of Layers

**Figure 16:**
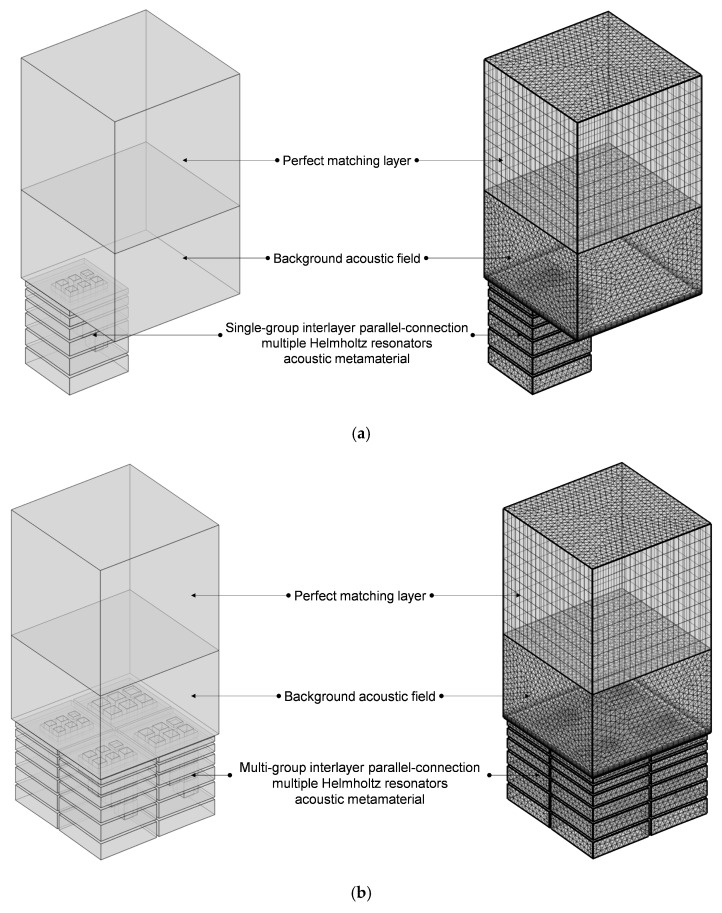
The finite element simulation of IPC–MHR acoustic metamaterial with 6 layers. (**a**) The single-group IPC–MHR; and (**b**) the multi-group IPC–MHR.

**Figure 17:**
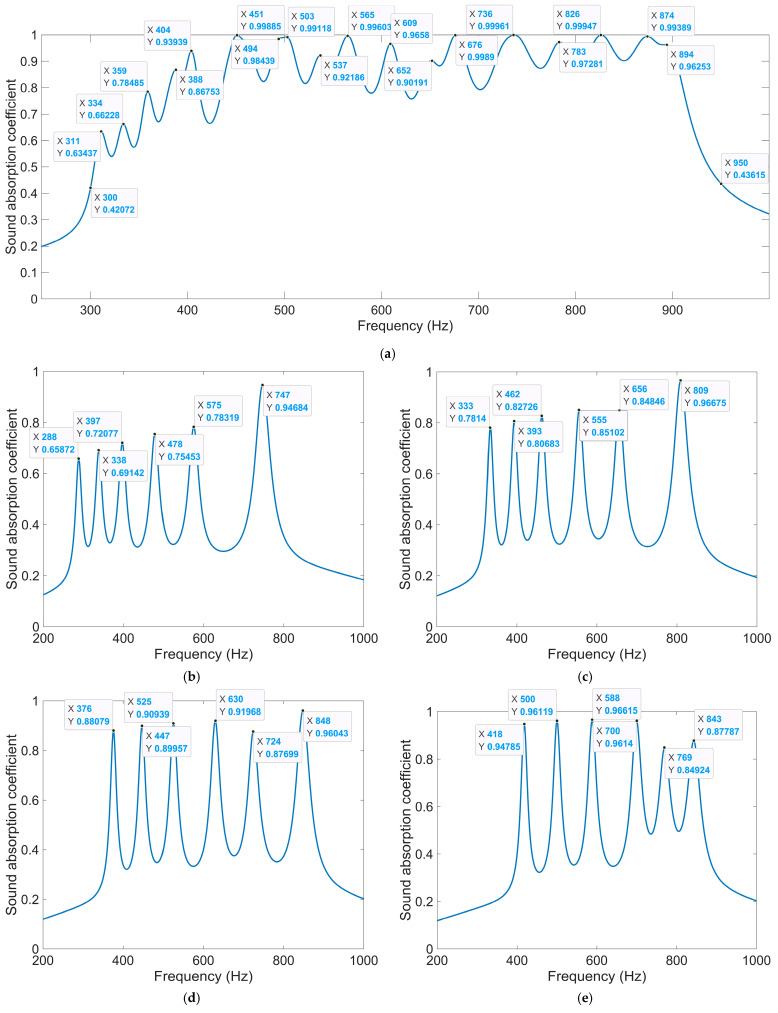
The distribution of optimal sound absorption property for the expected frequency range of 300–950 Hz. (**a**) The overall sound absorption performance; (**b**) the first module; (**c**) the second module; (**d**) the third module; and (**e**) the fourth module.

Furthermore, the number of layers in IPC–MHR can be altered as well to enlarge the sound absorption frequency band. The finite element simulation of IPC–MHR acoustic metamaterial with s layers is shown in Figure 16, and its total thickness is limited to 64 mm, which is consistent with the IPC–MHR in Figure 8 and Figure 14. Meanwhile, the side length of the square incident aperture for the four modules is set as 6 mm, 7 mm, 8 mm, and 9 mm, and the thicknesses of six layers are optimized by the particle swarm optimization algorithm. Supposing the expected frequency range is 300–950 Hz, the obtained optimal thicknesses of six layers are 15.6 mm, 24.9 mm, 33.7 mm, 43.1 mm, 52.4 mm, and 62 mm, respectively, and the corresponding average sound absorption coefficient reaches 0.8454. The distribution of optimal sound absorption property of the IPC–MHR with six layers is shown in Figure 17, the characteristics of which are consistent with those in Figure 15. The results indicate that the proposed IPC–MHR can gain excellent sound absorption in the low-frequency range with a wide frequency band.

**Figure 18:**
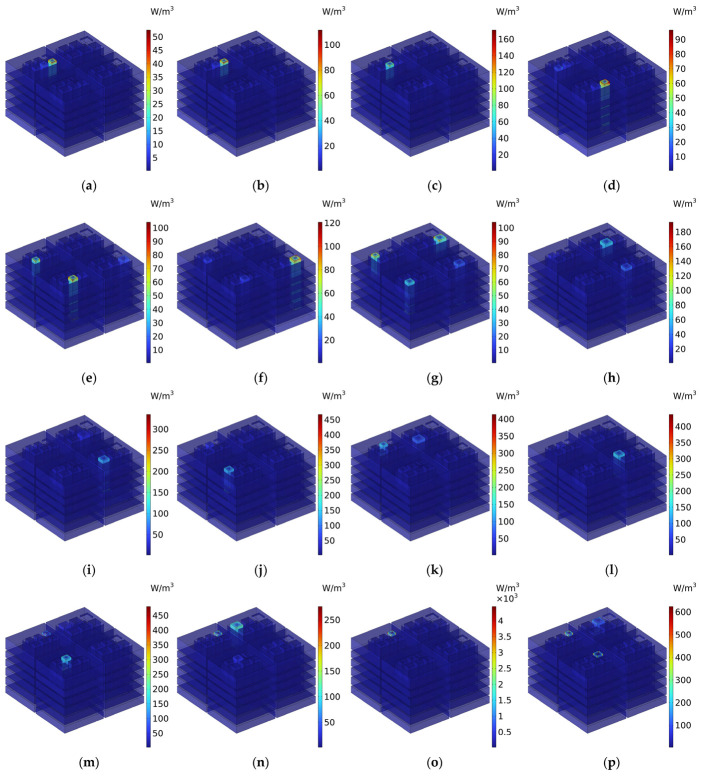

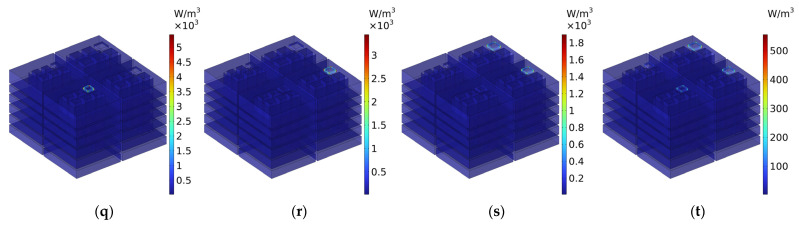
The distributions of the viscous power densities at the 20 marked frequency points in Figure 17a: (**a**) 300 Hz; (**b**) 311 Hz; (**c**) 334 Hz; (**d**) 359 Hz; (**e**) 388 Hz; (**f**) 404 Hz; (**g**) 451 Hz; (**h**) 494 Hz; (**i**) 503 Hz; (**j**) 537 Hz; (**k**) 565 Hz; (**l**) 609 Hz; (**m**) 652 Hz; (**n**) 676 Hz; (**o**) 736 Hz; (**p**) 783 Hz; (**q**) 826 Hz; (**r**) 874 Hz; (**s**) 894 Hz; and (**t**) 950 Hz.

To further demonstrate the sound absorption mechanism of IPC–MHR proposed in this study intuitively, the distributions of viscous power densities at the 20 marked frequency points in Figure 17a are obtained, as shown in Figure 18. It can be further demonstrated that the sound absorption effect of the IPC–MHR acoustic metamaterial is mainly realized by the resonance effect of each resonator and peripherally assisted by the coupling effect among different resonators, which are consistent with the characters of other acoustic metamaterials generated from the classic Helmholtz resonator. By expanding the space in the depth direction to increase the number of Helmholtz resonators and ensure sufficient volume for all the rear chambers in the proposed IPC–MHR, this novel acoustic metamaterial can achieve a large bandwidth and high sound absorption efficiency in the low-frequency range simultaneously.

## 5. Conclusions

The novel IPC–MHR acoustic metamaterial is proposed in this research to obtain optional sound absorption in the low-frequency domain, and its sound absorption property is investigated by the acoustic finite element simulation and standing wave tube measurement. The major achievements obtained in this study are as follows.

(1) The proposed IPC–MHR acoustic metamaterial consists of several layers, and the Helmholtz resonators among different layers are connected in parallel, which settles the conflict between the number of Helmholtz resonators and the volume of the rear cavity for each chamber with the given front area of the single-layer acoustic metamaterial.
(2) The sound absorption property of IPC–MHR acoustic metamaterial is studied by finite element simulation and further optimized by particle swarm algorithm. The average sound absorption coefficient in the discrete frequency band [200 Hz, 300 Hz] U [400 Hz, 600 Hz] U [800 Hz, 1250 Hz] is 0.7769 for the IPC–MHR with four layers. Through the optimization of the thickness of each layer, the average sound absorption coefficient in 250–750 Hz is up to 0.8068. Similarly, the optimized IPC–MHR with six layers gains an average sound absorption coefficient of 0.8454 in 300–950 Hz, which exhibits an excellent sound absorption performance in the low-frequency range with a wide frequency band.
(3) The sound absorption mechanism of IPC–MHR proposed in this research is displayed intuitively by the distributions of the viscous power densities for representative frequencies, which proves that the final absorption effect is mainly realized by the resonance effect of each resonator and assisted by coupling effect among different resonators.
(4) The actual sound absorption performance of IPC–MHR is validated by standing wave tube measurement with the sample fabricated through the additive manufacturing method. The consistency between the simulation result and the experimental data is satisfactory, which can prove the effectiveness of this IPC–MHR and the accuracy of the finite element simulation model.

The proposed IPC–MHR acoustic metamaterial can achieve optional excellent sound absorption performance in the low-frequency range with a wide frequency band, which is helpful to promote its practical application in the field of noise control.

## Figures and Tables

**Table 1:** The structural parameters for the IPC–MHR acoustic metamaterial are in Figure 5.

Meanings	Symbols	Value or Range
The total thickness of the IPC–MHR acoustic metamaterial	T_4_ + t_0_	64 mm
The thickness of all the wall	t_0_	2 mm
The side length of the cross-sectional shape for the multi-group IPC–MHR	S_0_	100 mm
The side length of the cross-sectional shape for the single-group IPC–MHR	(S_0_ − 3t_0_)/2	47 mm
The total thickness of the first layer	T_1_ = 3t_0_	6 mm
The length of the front aperture for the first layer	t_0_	2 mm
The thickness of the rear chamber for the first layer	T_1_–t_0_	4 mm
The total thickness of the second layer	T_2_ = 2T_1_ + t_0_	14 mm
The length of the front aperture for the second layer	T_1_	6 mm
The thickness of the rear chamber for the second layer	T_2_–T_1_–t_0_	6 mm
The total thickness of the third layer	T_3_ = 2T_2_ + t_0_	30 mm
The length of the front aperture for the third layer	T_2_	14 mm
The thickness of the rear chamber for the third layer	T_3_–T_2_–t_0_	14 mm
The thickness of the fourth layer	T_4_ = 2T_3_ + t_0_	62 mm
The length of the front aperture for the fourth layer	T_3_	30 mm
The thickness of the rear chamber for the fourth layer	T_4_–T_3_–t_0_	30 mm
The side length of the square incident aperture for the single-group IPC–MHR	a	[3 mm, 10 mm]
The side length of the square incident aperture for the multi-group IPC–MHR	a_i_	6 mm, 7 mm, 8 mm, 9 mm

**Table 2:** The utilized finite element simulation parameters for IPC–MHR acoustic metamaterial.

Parameters	Value or Type	Parameters	Value or Type
The type of mesh	Extremely fine mesh	The type of acoustic field	Plane wave
The type of grid	Free tetrahedral grid	The amplitude of background field	1 Pa
The selected solver	Steady state solver	The direction of incident wave	(0, 0, −1)
The relative tolerance of solver	0.001	The thickness of background field	64 mm
The maximum unit size	4 mm	The thickness of perfect matching layer	96 mm
The minimum unit size	0.04 mm	The equilibrium pressure	1 atm
The maximal unite growth rate	1.3	The equilibrium temperature	293.15 K
The curvature factor	0.2	The number of layers in distribution	10
The resolution of narrow region	1	The number of layers in boundary	10
The investigated frequency range	50–1300 Hz	The stretch factor in boundary	1.2
The frequency sampling interval	1 Hz	The regulation factor for thickness	1

## Data Availability

The original contributions presented in this study are included in the article.

## Appendix A

The symbols used in this study and their corresponding meanings are summarized in Table A1.

**Table A1:** The symbols used in this study and their corresponding meanings.

Symbols	Meanings
α	Sound absorption coefficient
*Z_total_*	The total acoustic impedance of the IPC–MHR acoustic metamaterial
*Z*_0_	The acoustic impedance of air under the condition of normal temperature and pressure
t_0_	The thickness of all the wall
S_0_	The side length of the cross-sectional shape for the multi-group IPC–MHR
T_1_	The total thickness of the first layer
T_2_	The total thickness of the second layer
T_3_	The total thickness of the third layer
T_4_	The thickness of the fourth layer
a	The side length of the square incident aperture for the single-group IPC–MHR
a_i_	The side length of the square incident aperture for the multi-group IPC–MHR
